# Giant nonequilibrium fluctuations in dilute and semidilute polymer solutions subjected to large temperature gradients

**DOI:** 10.1140/epje/s10189-026-00591-x

**Published:** 2026-06-02

**Authors:** J. Kantelhardt, D. Zapf, W. Köhler

**Affiliations:** 1 Physikalisches Institut, Universität Bayreuth, 95440 Bayreuth, Germany; 2 DLR-Institut für Vernetzte Energiesysteme, Deutsches Zentrum für Luft- und Raumfahrt (DLR), 70563 Stuttgart, Germany

## Abstract

**Abstract:**

Shadowgraph experiments have been performed on giant nonequilibrium fluctuations in solutions of polystyrene in toluene with polymer molar masses between 2.1 and 90.9 kg/mol and mass fractions ranging from 0.002 up to 0.6. Due to the large Soret coefficient of the polymer and the applied temperature difference of 50 K, a linear model is not sufficient to describe the time-dependent and static structure functions. Nonlinearities stemming mainly from the highly nonlinear concentration profile, as well as from the temperature and concentration dependence of various thermophysical parameters, are taken into account using a previously developed layer model. This model enables a detailed analysis of the signal generation within the shadowgraph cell. The thermal structure function mainly emerges from the hot top plate. For short polymer chains and/or low concentrations, the solutal structure function is dominated by the cold side. However, due to the complicated interplay between the Soret effect, the viscosity, and the gravitational quench, this can change for long chains and high concentrations, with the strongest solutal signal emerging from the hot side. Situations involving a non-monotonous layer sequence are also possible. The simulated structure functions agree reasonably with experimental data.

**Graphical Abstract:**

Dispersion of the structure functions due to nonlinearities in strong temperature gradients
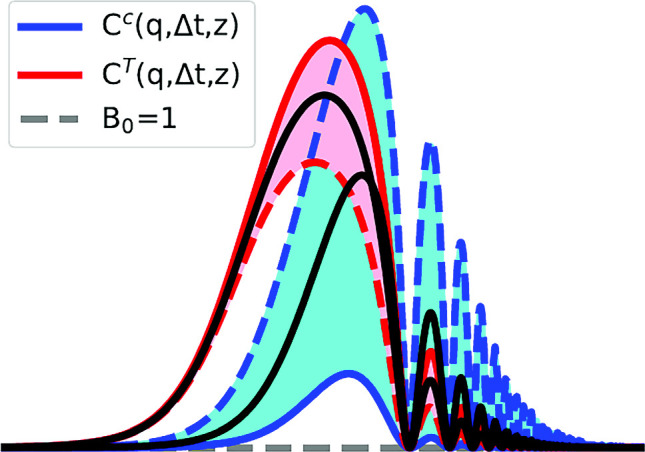

## Introduction

Thermodynamic nonequilibrium fluctuations of the velocity field in fluid mixtures couple to concentration and temperature gradients, making them visible in optical light scattering experiments [1]. They represent a new class of giant nonequilibrium fluctuations (NEFs) whose amplitudes exceed those of their equilibrium counterparts by orders of magnitude. In the absence of gravity, the NEFs decay by diffusion. Their static structure factor grows with a power law $\sim q^{-4}$ for small wave vectors *q* and is eventually limited only by the finite sample size [2, 3]. The stratification of the fluids in the imposed temperature and concentration gradients leads to a density gradient. Under the influence of gravity, volume elements transported by the fluctuations into an adjacent layer experience buoyancy, providing an additional relaxation path. For large length scales, i.e., small wave numbers, the relaxation by buoyancy surpasses the relaxation by diffusion and becomes the dominant mechanism [4].

The diffusive relaxation of NEFs, as observed at higher *q*-values, can be used to determine thermal diffusivities and mass diffusion coefficients of liquid mixtures. Under gravity, the crossover from diffusive to buoyancy-driven relaxation allows for the determination of the Soret and thermodiffusion coefficients [5, 6].

The study of NEFs in complex fluids under microgravity conditions aboard the International Space Station (ISS) is the objective of ESA’s GIANT FLUCTUATIONS project. Polymer solutions are among the envisaged samples, and the effects of high concentrations with overlapping chains, or even the proximity of the glass transition, as well as the effects of strong gradients are among the problems to be tackled.

In a previous study [7], we have investigated nonlinearities in shadowgraph experiments on NEFs resulting from strong temperature and concentration gradients in a dilute polymer solution. These nonlinearities originate both from nonlinear terms in the thermodiffusion equation and from the temperature and concentration dependence of the diffusion and thermodiffusion coefficients as well as a number of additional thermophysical parameters of the mixture. The measured shadowgraph structure functions can be described in terms of a superposition of signals originating from a series of layers stratified perpendicular to the temperature and concentration gradient. A linear description holds, and the extracted apparent Soret coefficient $S_T$ is approximately the same as the one for the mean sample temperature as long as, as a rule of thumb, the temperature difference across the sample stays well below the inverse of $S_T$.

In this paper, we extend the investigation to the semidilute and concentrated regimes and to different molar masses. The system is again polystyrene (PS) in toluene (Tol), as PS/Tol solutions will be investigated in the upcoming GIANT FLUCTUATIONS microgravity experiments. In addition, this is the only polymer solution for which the Soret coefficient is known as a function of molar mass, temperature, and concentration from the dilute up to the concentrated regime [8, 9], making it an ideal candidate for a quantitative mathematical modeling of the experiments.

The deconvolution of the measured shadowgraph signal into the contributions from the concentrations and corresponding Soret coefficients in the different layers is a highly complex inverse problem involving an integral equation that is practically unsolvable. The integrand itself is derived from a solution of the thermodiffusion equation for the given distribution of Soret coefficients. To circumvent this problem, we use a different approach and simulate the measured shadowgraph structure functions based on the known concentration and temperature dependencies of all parameters and compare them with the experimental results.

## Experiment

*Setup* Experiments were performed using the shadowgraph setup described in Refs. [7, 10] with only minor modifications. The shadowgraph cell consists of two sapphire windows separated by a Teflon spacer of $h=5\,\hbox {mm}$. The free aperture of the cell is $13\,\hbox {mm}$. Peltier elements with a central hole (AMS Technologies, TB-109) in combination with two electrical control units (LFI’s, Wavelength Electronics, LFI-3751) with an accuracy of $\Delta T\pm 0.01\text {K}$ allow to maintain a prescribed temperature difference of $\Delta T = 50\,\hbox {K}$ across the fluid. In the experiments reported here, the mean sample temperature was $T_0 = 298\,\hbox {K}$. The lower and the top plates were kept at $T_1 = 273\,\hbox {K}$ and $T_2 = 323\,\hbox {K}$, respectively. The sample is illuminated with the parallel beam of a superluminescent diode (Superlum, SLD-261-MD-670, 670 nm), expanded to 18.2 mm in diameter. The transmitted beam is detected by a CMOS camera (Hamamatsu Orca-Fusion Digital Camera C14440).

*Samples* Tol from VWR Chemicals with a purity of 99.9% was used as solvent. The polystyrene polymers (SEC calibration standards) were obtained from PSS-Agilent Technologies with weight average molar masses of $M_w = 2.090\,\hbox {kg/mol}$ (2.1k), $4.840\,\hbox {kg/mol}$ (4.8k), $17.900\,\hbox {kg/mol}$ (17.9k), and $90.900\,\hbox {kg/mol}$ (90.9k) and narrow molar mass distributions.

## Theory

### General picture

As a starting point, we briefly summarize the layer model for the treatment of nonlinearities that we have developed in Ref. [7].

In shadowgraph experiments on binary liquid mixtures, the sample is subjected to a vertical temperature gradient. A superimposed concentration gradient builds up due to the Soret effect. Its steady-state amplitude is described by the stationary thermodiffusion equation

1 $$\begin{aligned} \nabla c + S_T c(1-c) \nabla T = 0 \end{aligned}$$

for the concentration *c* (mass fraction) of the independent component, which in this case is the polymer.

So far, data evaluation of Soret experiments has usually been based on a linearization of Eq. (1), where the term $c(1-c) = c_0(1-c_0)$ is taken as constant with $c_0$ being the mean concentration [5]. At the same time, the sample temperature is given by the mean sample temperature $T_0$. All parameters of the mixture, as well as the temperature and concentration gradients, are taken as constant at $c=c_0$ and $T=T_0$. The computed transport coefficients are assumed to represent the ones at this fixed concentration and temperature.

In the presence of strong gradients, the linear approximation is no longer applicable. The temperature dependence of the sample parameters and, most importantly, the nonlinear term in the thermodiffusion equation lead to position-dependent properties and nonlinear temperature and concentration profiles. The concentration dependence is of negligible importance for very dilute polymer solutions, but it becomes a decisive factor in the semidilute and concentrated cases reported here.

What is the general scenario when the polymer concentration is increased? The Soret and the mutual diffusion coefficients of polymer solutions are strongly composition dependent and follow molar mass independent scaling laws above the overlap concentration $c^*$ [11]. At the same time, the chain entanglements in the semidilute regime lead to a strong increase in the macroscopic shear viscosity as described by de Gennes’ reptation model [12, 13]. However, this does not yet lead to a significant change in local friction on the length scale of a segment: both the thermodiffusion coefficient and the solvent self-diffusion coefficient approximately retain their dilute solution values at semidilute concentrations [14]. Driven by the growing osmotic modulus, the mutual diffusion coefficient increases and the Soret coefficient decreases with increasing polymer concentration [11].

As the polystyrene concentration is increased further into the concentrated regime, the glass transition temperature $T_g$ of the solution, which grows from $T_g^\textrm{tol} = 117\,\hbox {K}$ for pure toluene [15] to $T_g^\textrm{ps} \approx 363\,\hbox {K}$ for pure polystyrene [16], comes closer to room temperature. The glass transition is thus approached along the concentration axis [11, 17]. In this regime of the supercooled liquid, the local dynamics of the monomers and solvent molecules slow down, enslaving all diffusion and thermodiffusion dynamics. Interestingly, the Soret coefficient remains unaffected by the glass transition [8].

Unfortunately, there are little data available on the non-isothermal transport coefficients of polymer solutions. The PS/Tol system that we are studying here is, to our knowledge, the only one for which the diffusion, thermodiffusion, and Soret coefficients are known as a function of molar mass *M*, polymer mass fraction *c*, and temperature *T*. This provides us with a unique opportunity to model the shadowgraph experiment within our nonlinear layer framework and compare the results with those of actual experiments.

### The shadowgraph experiment

The Soret cell in the shadowgraph experiment consists of two sapphire windows parallel to the (*x*, *y*)-plane with a spacing *h* along the *z*-direction, which is anti-parallel to the direction of gravity. The lower plate, at $z=0$, has a temperature $T=T_1$, and the upper plate at, $z=h$, has one of $T=T_2 > T_1$. The mean temperature $T_0 = (T_2 + T_1)/2$ is approximately, but not precisely, in the midplane of the cell [7]. The basic assumption of the layer model is that all parameters can be taken as constant within a thin layer perpendicular to the temperature gradient but not along the *z*-direction.

Shadowgraph experiments are evaluated on the basis of the differential dynamic analysis (DDA) [5, 18–24]. The light scattered by NEFs in a thin liquid layer is recorded at a distance *Z* from the center of the layer. The structure function

2 $$\begin{aligned} C(q, \Delta t) = 2 \tilde{T}(q) S(q) [1-f(q,\Delta t)] + B(q) \end{aligned}$$

is computed from Fourier-transformed difference images in *q*-space [5]. Here, $\tilde{T}(q) = 4 \sin ^2(q^2\,Z/(2k))$ is an optical transfer function [18], *B*(*q*) is a background term, and *S*(*q*) is the static structure factor of the fluctuations.

3 $$\begin{aligned} f(q,\Delta t) = \frac{S^T(q)}{S(q)}\exp \left( -\frac{\Delta t}{\tau ^T}\right) + \frac{S^c(q)}{S(q)}\exp \left( -\frac{\Delta t}{\tau ^c}\right) \end{aligned}$$

is the bimodal intermediate scattering function. The static structure factors $S^i(q) = {I_0^i}/({1 + (q/q^i_{ro})^{4})}$ of the thermal ($i=T$) and the solutal ($i=c$) NEFs add up to the total static structure factor $S(q) = S^T(q) + S^c(q)$. Both diverge for large wavevectors proportional to $q^{-4}$, whereas they are cut off by gravity (gravitational acceleration *g*) below their respective roll-off wavevectors [25]

4 $$\begin{aligned} q^T_{ro} = \left( \frac{\beta _T g \nabla T }{\nu D_{th}}\right) ^{1/4} \textrm{and}~~~ q^c_{ro}= &   \left( \frac{\beta _c g \nabla c }{\nu D}\right) ^{1/4}. \end{aligned}$$

Here, *D*, $D_{th}$, and $\nu$ are the Fickian diffusion coefficient, the thermal diffusivity, and the kinematic viscosity, respectively. The thermal and solutal time constants are given by [26]

5 $$\begin{aligned} \tau ^T(q)= &   \frac{1}{D_{th} q^2 (1 + (q^T_{ro}/q)^4)} \end{aligned}$$

6 $$\begin{aligned} \tau ^c(q)= &   \frac{1}{D q^2 (1 + (q^c_{ro}/q)^4)} ~. \end{aligned}$$

The amplitudes are

7 $$\begin{aligned} I_0^T\propto &   \frac{(\partial n/\partial T)_{p,c}^2 (\nabla T)^2}{D_{th} \nu } \left( \frac{1}{q_{ro}^T}\right) ^4 \end{aligned}$$

8 $$\begin{aligned} I_0^c\propto &   \frac{(\partial n/\partial c)_{p,T}^2 (\nabla c)^2}{D \nu } \left( \frac{1}{q_{ro}^c}\right) ^4 ~. \end{aligned}$$

The partial derivatives of the refractive index account for the optical contrast of the NEFs [27].

### Formulation of the layer model

In the layer model, the measured structure function of a thick sample is calculated as a linear superposition of the structure functions (Eq. (2)) emerging from the infinitely thin stratified layers at vertical positions *z*. Thus, Eq. (2) is replaced by [7]

9 $$\begin{aligned} C(q,\Delta t) = \frac{1}{h} \int _0^h 2 \tilde{T}(q,z) S(q,\Delta t,z) dz + B_0 ~.\end{aligned}$$

This equation is valid as long as the fluctuations in the *z*-direction are uncorrelated, which holds for the entire experimentally accessible *q*-range, possibly except the very low-*q* end. Details are discussed in Ref. [7]. In order to compute $C(q,\Delta t)$, it is necessary to know the temperature and concentration distributions within the cell, i.e., how they depend on the vertical position *z*.

Since the heat conductivity $\kappa (T)$ of PS/Tol solutions depends only weakly on composition, we approximate the one of the mixture by the one of pure toluene as reported by Kashiwage et al. [28]: $\kappa (T) = k_0 + \alpha (T-T_0) = [ 0.1307 - 2.88\times 10^{-4}\hbox {K}^{-1} \times (T-T_0) ] \hbox {W}/{\hbox {m}\,\hbox {K}}$. This results in a weakly nonlinear temperature profile within the cell [7]:

10 $$\begin{aligned} \frac{T(z) - T_1}{\Delta T} = \frac{-1 + (1 + 2 \beta \xi + \beta ^2 \xi )^{1/2}}{\beta } \end{aligned}$$

Here, $$T_0=298.15\,\hbox {K}$$, $$\beta = \alpha \Delta T / k_0$$, $$\xi = z/h$$, and $$T_1 = T(z=0)$$.

For the calculation of the concentration profile *c*(*z*), we resort to the parameterization of the Soret coefficient $S_T(M,c,T)$, which has been described in Ref. [9] on the basis of room-temperature measurements in Refs. [8, 11], covering a PS molar mass range from $4.75\,\hbox {kg/mol}$ up to $4060\,\hbox {kg/mol}$ and concentrations from the dilute limit $c \rightarrow 0$ up to the highly concentrated regime with $c=0.87$. These experiments were later complemented with temperature-dependent measurements over a temperature range from $293\,\hbox {K}$ to $343\,\hbox {K}$, resulting in [9]

11 $$\begin{aligned} S_T(M,c,T)= &   \frac{a}{1 + b\, c\,^\beta } \left( \frac{T'_0}{T} \right) ^{2.4}~. \end{aligned}$$

Here, $T'_0 = 295\,\hbox {K}$, $a = 3.294\times 10^{-4}\, M^{0.58}$, $\beta = 0.82 + 35.42\, M^{-0.5}$, and $b = ({a}/{0.012}) - 1$, with *M* being the molar mass in kg/mol.

Equation (1) can be solved numerically with $S_T(M,c,T)$ from Eq. (11) after separation of variables. The integration constant $c(z=0)$ is obtained from the condition of mass conservation. The result is the concentration *c*(*z*) for each layer at height *z* as detailed in Ref. [7].

In addition to the temperature and concentration distributions and their respective gradients, a number of additional thermophysical parameters are needed to model the structure function $C(q,\Delta t)$ according to Eq. (9). Most of these parameters exhibit only a weak dependence on concentration and/or temperature, which can be linearized or approximated by their dilute solution values. In particular, the temperature dependence can frequently be neglected. A detailed derivation can be found in Ref. [7]. Here, we only briefly report the numbers used for our simulations.

The density of the solution is linearized both in temperature and concentration: $\rho (c,T) = [ 863 + 182 \, c - 0.933{\hbox {K}^{-1}} \, (T-T_0) ] {\,\hbox {kg}\, \hbox {m}^{-3}}$. Since higher-order terms are negligible, the thermal and solutal expansion coefficients are constant. Their values are $\beta _T = \frac{1}{\rho }( {\partial \rho }/{\partial T})_{p,c} = -1.08 \times 10^{-3}{\hbox {K}^{-1}}$ and $\beta _c = \frac{1}{\rho }\left( {\partial \rho }/{\partial c} \right) _{p,T}= 0.210$, respectively.

The modeling of the viscosity [7] is based on the truncated Martin’s equation [29] for the specific viscosity: $\eta _{sp} = \eta /\eta _0 -1 = \tilde{c}[\eta ] ( 1 + K_H \tilde{c}[\eta ] + \frac{1}{2} (K_H \tilde{c}[\eta ])^2 + \frac{1}{6} (K_H \tilde{c}[\eta ])^3 )$. The intrinsic viscosity $[\eta ]$ is measured in mL/g and the concentration $\tilde{c} = c \, \rho \times 10^{-3}$ in g/mL. The Huggins constant is $K_H \approx 0.4$. The approximation gives a good description [29] up to at least $\log (\tilde{c}[\eta ]) = 2$ (the ‘$\log$’ is missing in Ref. [7]), which holds for all of our samples. The intrinsic viscosity is approximated by an improved alternative to the Kuhn–Mark–Houwink–Sakurada equation [30]: $\log [\eta ] = -0.538 + 0.203 (\log M) + 0.0471 (\log M)^2$. The viscosity of toluene, $\eta _0$, is described by standard reference data of Santos et al. [31]: $\ln \eta ^* = -5.2203 + {8.964}/{T^*} - {5.834}/{(T^*)^2} + {2.089}/{(T^*)^3}$. The reduced variables are $T^*=T/T_0$ and $\eta ^* = \eta _0(T) / \eta _0(T_0)$ with $T_0 = 298.15\,\hbox {K}$ and $\eta _0(T_0) = 554.2\,{\mu Pa\,s}$. Finally, the dynamic and kinematic viscosities of the polymer solution are obtained as $\eta (c,T) = \eta _0(T)(\eta _{sp}(c) + 1)$ and $\nu (c,T) = \eta (c,T) / \rho (c,T)$, respectively.

The weak concentration dependence of the thermal diffusivity can be neglected, and its linear temperature dependence is given by $D_{th} = [8.3 - 2.5 \times 10^{-2} (T-T_0)] \times 10^{-8}{{\hbox {m}^2}{s}}$ [7]. The Fickian diffusion coefficient is obtained from Eq. (11) in combination with a suitable parameterization of the thermodiffusion coefficient $D_T$ as $D = D_T/S_T$. This work uses higher concentrations, for which appreciable decay of $D_T$ has already occurred. The dilute limit approximation from Ref. [7] is no longer valid in this case. The slowing down of thermodiffusion with increasing polymer concentration, which parallels the slowing down of the solvent self-diffusion coefficient [14], is identified in Refs. [8, 11] as a finger print of the approaching glass transition with increasing polymer concentration. In order to cope with this pronounced concentration dependence, we have fitted a third-order polynomial in *c* to the concentration-dependent measurements of $D_T$ in Ref. [8] over the concentration range from the dilute limit up to $c=0.87$:

12 $$\begin{aligned} D_T(c,T)= &   [1.09 - 1.52 c - 0.29 c^2 + 0.71 c^3 \nonumber \\  &   + 0.0073 (T-T_0)] \times [10^{-11}]{\frac{m^2}{sK}} \end{aligned}$$

The temperature dependence has been taken from Ref. [32].

## Results and discussion

*Concentration distribution*

**Fig. 1:**
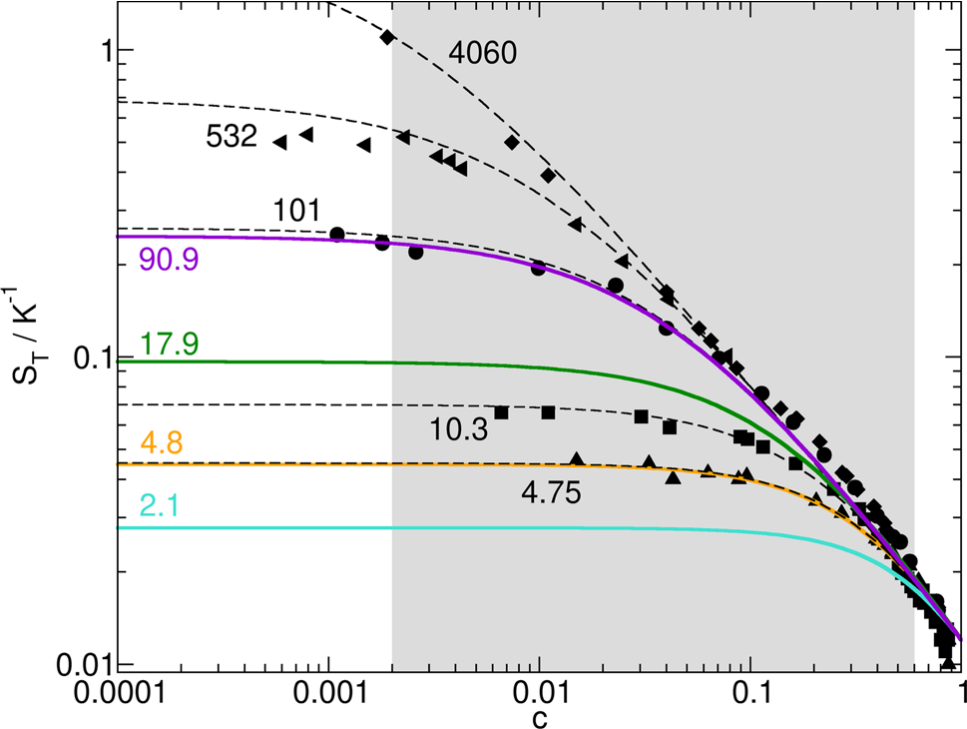
Concentration dependence of $S_T$ of PS/Tol solutions as a function of polymer concentration *c* for various molar masses (in kg/mol). The experimental data are from Ref. [8] at $T=295\,\hbox {K}$ with fits of Eq. (11) (dashed lines). The colored lines are calculated Soret coefficients for the polymers used in this work with an only slightly different reference temperature of $T_0 = 298\,\hbox {K}$. The shaded area marks the concentration range covered in this study

As reflected by Eq. (11), the Soret coefficient of a polymer solution depends strongly on both the molar mass and the concentration of the polymer [8, 11, 33]. The dilute solution value of $S_T$ in a good solvent grows in proportion to $M^{\nu }$, with $\nu \approx 0.58$ being the Flory scaling exponent. For high polymers, it reaches values that exceed the Soret coefficient of small molecules by orders of magnitude. The Soret coefficient decreases as the polymer concentration increases throughout the semidilute regime, eventually reaching values characteristic of small molecules at very high polymer concentrations. Figure 1 shows experimental data for the Soret coefficient of various PS molar masses from Ref. [8] with fits of Eq. (11). Included in the plot are the calculated concentration dependencies of $S_T$ for the four molar masses investigated in this work.

**Fig. 2:**
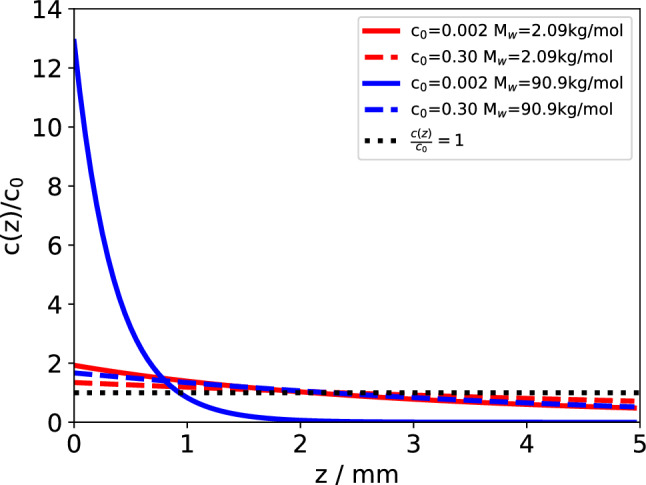
Vertical concentration distribution for a low ($c_0=0.002$) and a high ($c_0=0.3$) concentration of the shortest (2.1k) and a longest (90.9k) PS in Tol. The curves are normalized to the respective equilibrium concentration $c_0$

The variations of $S_T$ result in peculiar vertical concentration distributions in the cell that strongly depend on the concentration and the molar mass of the polymer. A few examples are plotted in Fig. 2 for a low ($c=0.002$) and a high ($c=0.3$) concentration of both the shortest (2.1k) and the longest (90.9k) polymer studied. The temperature difference is $\Delta T = 50\,\hbox {K}$.

The relative concentration shifts of the short polymer are not very sensitive to the mean concentration and amount to approximately a factor of two to three between the cold wall at $z=0$ and the hot wall at $z=h=5\,\hbox {mm}$. Remarkably, the behavior of the long polymer with the high concentration is very similar to the short polymer, which is a direct consequence of the small Soret coefficient of the entangled solution at high concentrations.

The situation changes completely for the dilute solution of the long polymer (90.9k, $c=0.002$). Because of its large Soret coefficient, almost all of the polymer is drawn toward the cold wall, leaving hardly any in the top (warm) half of the cell. Without the self-limiting effect of the decreasing Soret coefficient with increasing concentration near the cold wall, polymer accumulation on the cold side would be even more extreme.

*Structure functions*

In this section, we discuss the structure functions obtained from the layer model exemplarily for a short and a long polymer both in the dilute limit and at high concentrations.

It is not possible to distinguish signal contributions from individual layers experimentally. Measurements only yield an integral signal, which is a linear superposition over the height of the cell. In order to allow for a comparison with experiments, we simulate the signal by averaging over the contributions from all layers according to Eq. (9). This simulated structure function is directly comparable to an actual measurement and can be treated in the same way for further data evaluation. At the same time, the simulation provides the structure functions of the individual layers, which can be used to analyze the inherent polydispersity of the measurements. To align the simulated and measured amplitudes, a phenomenological scaling factor was introduced. Individual adjustments to each measurement indicated a value around 10 in most cases, with a few outliers reaching up to 12. To ensure consistent treatment of all measurements, a constant value of 10 was chosen. Currently, we are unsure about the origin of this scaling factor, and further work is required to clarify this. While the plots shown here seem to suggest that the scaled simulations tend to overestimate the experimental results, this is not the case when all measurements are considered. We will discuss the simulations and measurements in detail below. While the achieved agreement is by no means perfect, we will demonstrate that it is good enough to show that the model captures the essential features of the nonlinearities in the experiments.

**Fig. 3:**
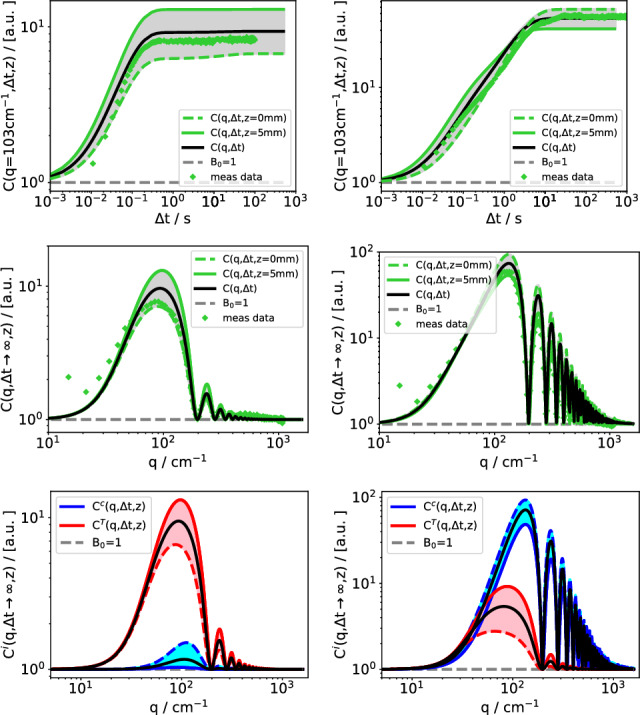
Simulated and measured structure functions of PS(2.1k) in Tol. Left column: $c=0.002$, right column $c=0.3$. Time-dependent structure function $C(q,\Delta t)$ (row 1) and static structure function $C(q,\Delta t \rightarrow \infty )$ (row 2). Experimental data (symbols) and simulation of measured structure functions (solid black line) are shown in rows 1 and 2. The green region shows the dispersion of the structure functions for the individual layers from the bottom ($z=0$, cold) to the top ($z=h$, hot) of the cell. Row 3 shows the separation of the simulated structure functions into the thermal and the solutal contributions. $T_0 = 298\,\hbox {K}$, $\Delta T = 50\,\hbox {K}$

**Fig. 4:**
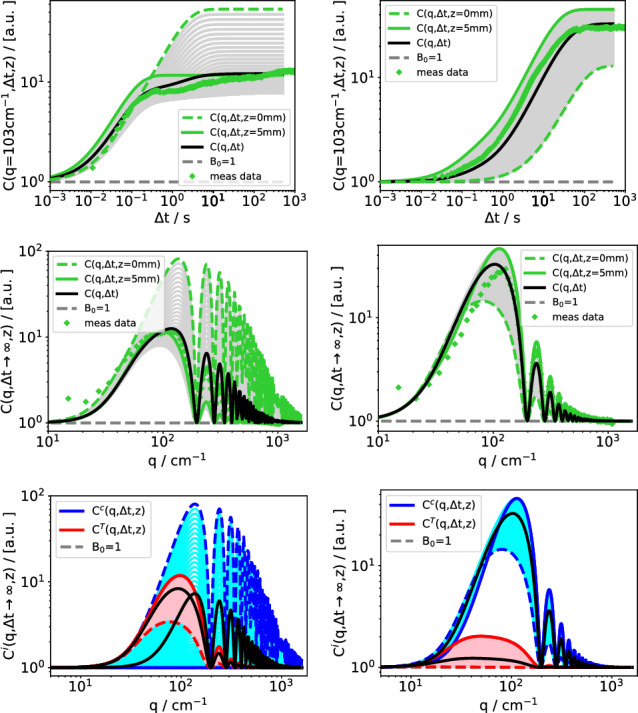
Same as in Fig. 3, however for PS(90.9k)

Figures 3 and 4 both show a $3 \times 2$ matrix of simulation plots. Figure 3 is for the low (2.1k) and Fig. 4 for the high molar mass (90.9k). The first column is for a low ($c=0.002$) and the second column for a high concentration ($c=0.3$). The first rows show time-dependent structure functions $C(q,\Delta t)$ for a selected wavevector of $q=103\,\hbox {cm}^{-1}$. The second rows contain the stationary structure functions $C(q,\Delta t \rightarrow \infty )$, and the third rows the decomposition of the latter into thermal and solutal contributions.

The simulated structure functions for the individual 100 layers are shown as a sequence of thin colored lines, which sometimes appear as a continuum in the plot. The layer adjacent to the cold wall ($z=0$) is represented by a dashed line and the one adjacent to the hot wall ($z=h$) by a solid colored line. The simulated measured structure functions are plotted as solid black lines. In addition to the simulations, rows 1 and 2 also contain experimental data points.

The first observation from Figs. 3 and 4 is the already mentioned decent agreement between the simulated structure functions and the experimental data, i.e., the symbols are reasonably close to the solid black lines in the 1st and 2nd row of both figures. The dispersion of the structure functions of the individual layers depends strongly on both the polymer molar mass and concentration, but in all cases it is considerable. A closer inspection reveals different characteristic scenarios.

The structure functions of the short polymer at low concentration (Fig. 3, 1st column) are essentially thermal with a very small solutal contribution. This is most clearly seen in the third row, where the static structure functions are separated into the thermal and the solutal modes (note that the *y*-axis is logarithmic). The signal from the hottest layer ($z=h$, thick solid line) is the strongest one during the entire evolution of the time-dependent structure function and, hence, also in the static structure function.

The situation is different for a high concentration of short polymers (Fig. 3, 2nd column). Here, the thermal mode dominates only at short times; at long times, the signal becomes mainly solutal. As can be seen in the third row, the solutal amplitude of the simulated static structure function exceeds the thermal amplitude by roughly one order of magnitude. The time-dependent structure function receives its main contribution at short times, when it is primarily thermal, from the layer adjacent to the hot wall at $z=h$. The solutal signal dominates at long times and emerges mainly from the layer next to the cold wall ($z=0$), as can be seen from the dashed line in the plot (Fig. 3, 1st row, 2nd column). The crossover occurs around $t\approx 2\,\hbox {s}$, where the signals from the hottest and the coldest layers have identical amplitudes and the dispersion becomes very narrow.

The domination of the layers next to the cold wall in the solutal signal is mainly a consequence of the positive Soret coefficient of PS in the solvent toluene, which leads to an accumulation of the polymer on the cold and a depletion on the hot side, as shown in the concentration distributions in Fig. 2.

In summary, the short polymer behaves similar to what one would expect for mixtures of small molecules. Typical polymer effects are not yet pronounced and the relevant thermophysical parameters, in particular the Soret coefficient and the viscosity, are similar to low-molar-mass mixtures.

The behavior changes for high molar masses of the polymer (Fig. 4). The large Soret coefficient of the dilute polymer leads to an exponential instead of a linear concentration distribution with almost all polymer on the cold side and a steep concentration gradient.

Already for concentrations as low as $c=0.002$, the solutal signal from the cold side exceeds the amplitude of the thermal one, whereas it practically vanishes on the hot side. As can be seen from the time-dependent structure function (Fig. 4, 1st row, 1st column), the major contribution to the total signal stems from the hot wall at short times and from the cold wall at long times. The layer with the smallest signal contribution is no longer at one of the walls but rather somewhere in between.

The entanglements at high polymer concentrations change the behavior of the solutal structure function completely. They reverse the ordering of the contributions to the solutal structure function, which is now strongest on the warm and weakest on the cold side, as shown in Fig. 4, 2nd column.

To understand this phenomenon, it is helpful to take a closer look at the amplitude of the solutal structure function. For large wave vectors with $(q/q^c_{ro})^4 \gg 1$, the static structure function can be written from Eqs. (1), (3), (4), and (8) in the form

13 $$\begin{aligned} S^c(q) \propto \frac{(\partial n/\partial c)_{p,T}^2 \nabla T^2}{q^4} \cdot \frac{\left( c(1-c)S_T \right) ^2}{D \nu } ~. \end{aligned}$$

The Soret effect results in an increase in concentration at the cold plate ($z = 0$). What effect does this have on the amplitude of the structure function of the adjacent layer?

The first factor in Eq. (13), which contains the optical contrast factor and the temperature gradient, depends only weakly on temperature and concentration and can be taken as constant. The second factor, however, consists of several counteracting contributions. The concentration prefactor $c(1-c)$ is bell-shaped. It increases linearly for small concentrations, flattens out around $c=0.5$, and then decreases again. The Soret coefficient $S_T$ significantly decreases above the overlap concentration, i.e., for the higher concentration of PS(90.9k) (see also Fig. 6). The diffusion coefficient in the denominator increases in the semidilute regime proportional to $1/S_T$. Around $c=0.3$, however, the approaching glass transition at high concentrations casts its shadow ahead and flattens the concentration dependence of *D* [8, 11]. Finally, the viscosity $\nu$ of the entangled solution of the long polymer chains shows a strong increase with *c*. As it appears in the denominator, it reduces the amplitude. The combined effect of the four contributions appearing in the second factor of Eq. (13) is the observed reduction of the amplitude of the solutal static structure factor on the cold side, despite the polymer accumulation due to the Soret effect. Conversely, polymer depletion on the hot side increases the solutal amplitude. The quantitative treatment requires to take not only the concentration but also the temperature dependencies into account, but this does not change the general picture discussed here for the high concentration of the longest polymer PS(90.9k).

For different molar masses and concentrations, the delicate balance of the four contributions can be different. In particular in the dilute limit, the concentration prefactor increases linearly with *c*, whereas $S_T$, *D*, and $\nu$ approximately retain their dilute solution values. This leads to the domination of the solutal signal from the cold side as observed for the low concentrations.

An additional unexpected effect arises from the layer-dependence of the gravitational quench, which becomes important at wavevectors around the first maximum of the optical transfer function *T*(*q*). In Fig. 4, 3rd row, 2nd column this refers to the range $q < 200\,\hbox {cm}^{-1}$. The major part of this maximum shows the same behavior as the higher maxima: the strongest contribution to the solutal static structure function originates from the hot side. On the left side, for $q < 60\,\hbox {cm}^{-1}$, the sequence is, however, reversed and the cold side dominates. The main cause for this inversion is the layer-dependence of the solutal roll-off wavevector $q_{ro}^c$.

**Fig. 5:**
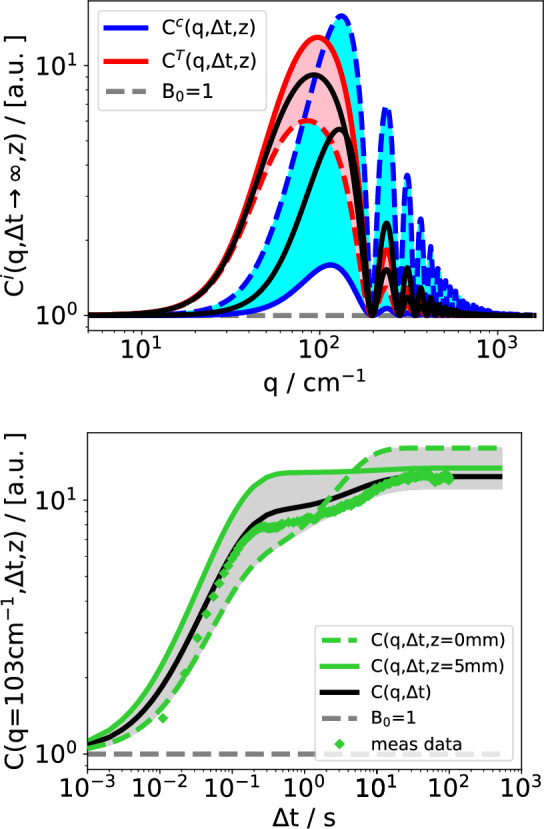
Simulated and measured structure functions of PS(4.8k) in Tol, $c=0.01$ for the individual layers. Top: Separation of the simulated static structure functions into the thermal and the solutal contributions. Bottom: Time-dependent structure function $C(q,\Delta t)$. The solid black lines represent the simulated measured structure functions. The symbols are experimental data. $T_0 = 298\,\hbox {K}$, $\Delta T = 50\,\hbox {K}$

Figure 5 illustrates a further example with a non-monotonous variation of the signal amplitude across the layers. The system is PS(4.8k) at one-percent mass fraction. The static structure functions show the generic behavior with a domination of the hot side in the thermal and the cold side in the solutal mode. The time-dependent structure function shows the known crossover from thermal at short times to solutal at long times. While the maximum of the steady-state amplitude is on the cold side at $z=0$, its minimum is not on the hot side but rather somewhere in between.

**Fig. 6:**
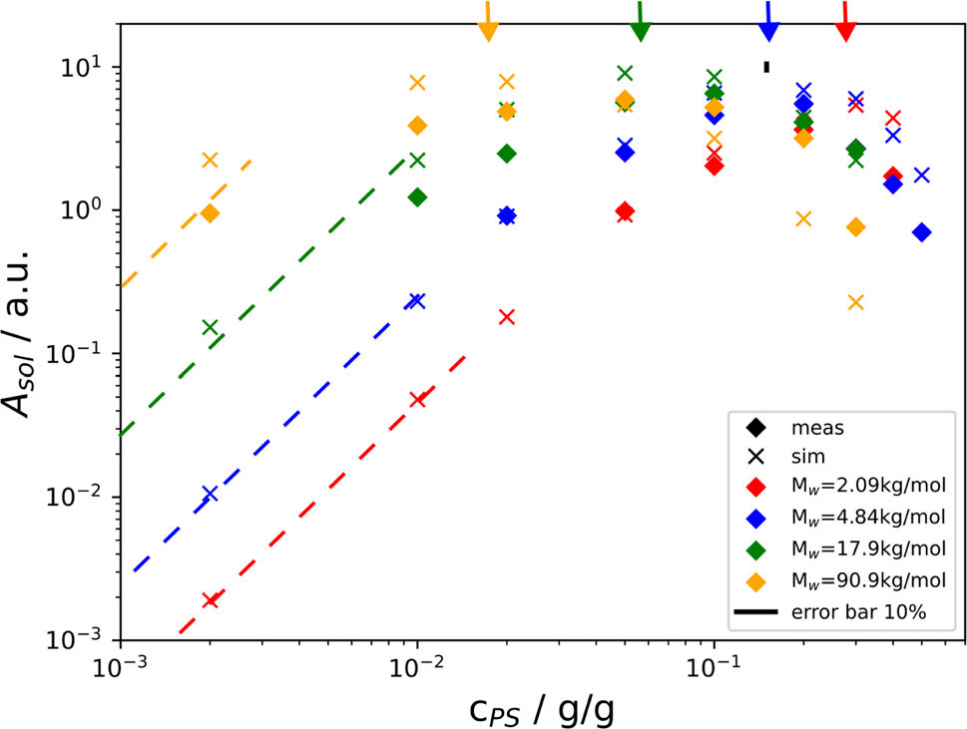
Simulated and measured amplitudes of the static structure functions at a wave vector of $q=367\,\hbox {cm}^{-1}$. The dashed straight lines in the dilute regime have a slope of 2 and are guides to the eye. The arrows on the top indicate the respective overlap concentrations $c^*$. $T_0 = 298\,\hbox {K}$, $\Delta T = 50\,\hbox {K}$

Finally, Fig. 6 summarizes the dependence of the solutal amplitude of the static structure function on the concentration up to $c=0.6$ for all investigated molar masses. The plot shows the amplitude at $q=367\,\hbox {cm}^{-1}$, which corresponds to the third maximum of the optical transfer function. This wavevector has been selected, because it is already significantly above the roll-off wavevector and the structure function still has a sufficiently large amplitude to avoid excessive noise. Again, the agreement between simulation and experiment is not perfect but acceptable.

The amplitudes clearly show a maximum and a pronounced decay at higher polymer concentrations. The concentration where the maximum is observed decreases with increasing polymer molar mass. It is roughly around the molar mass-dependent overlap concentration $c^*$ [8], which is the concentration where the chains start to entangle and the semidilute regime begins. The overlap concentrations for the four investigated molar masses have been estimated by interpolation of the values given in Ref. [8] with a scaling law $c^* \sim M^{1-3\nu }$. They are indicated in Fig. 6 by color-coded vertical arrows along the upper *x*-axis. The decrease of the Soret coefficient above $c^*$, as shown in Fig. 1, is a decisive contribution to the amplitude loss at higher polymer concentrations. The amplitudes grow proportional to $c^2$ in the dilute regime as predicted by Eqs. (4) and (8) for wave vectors above $q_{ro}^c$. This is indicated by the dashed straight lines.

## Summary and conclusion

We have analyzed the shadowgraph signals of polymer solutions with concentrations ranging from the dilute limit far into the semidilute and the beginning concentrated regime. The molar masses range from short chains with $M_w=2.090\,\hbox {kg/mol}$, corresponding to only about 20 monomers, up to high polymers with a molar mass of $M_w = 90.9\,\hbox {kg/mol}$ and almost $10^3$ repeat units.

This work aims to understand and model the shadowgraph structure functions under experimental conditions that extend well beyond the linear regime. Nonlinearity arises partly from the concentration and temperature dependence of several thermophysical parameters. The most significant impact stems, however, from the nonlinear terms in the thermodiffusion equation, resulting in substantial variation in the concentration gradient throughout the sample. These nonlinearities are important in situations where, as a rule of thumb, the product $S_T \Delta T$ exceeds unity. Due to the large Soret coefficient of dilute polymer solutions, this condition is easily met for moderate temperature differences and may even be difficult to avoid altogether. Since it was our intention to test the applicability of our model under strongly nonlinear conditions, we selected a large temperature difference of $\Delta T = 50\,\hbox {K}$.

It is no longer possible to evaluate the structure functions straightforwardly in order to determine, e.g., the Soret coefficient under these conditions. Instead, a previously developed layer model [7] was employed to model the measured structure function as a superposition of contributions emerging from a succession of thin layers parallel to the cell windows. Within the layers, but not from layer to layer, all thermophysical parameters are taken as constant. This enables both a quantitative modeling of the measured structure functions and a spatially resolved analysis throughout the entire sample. A reasonable level of agreement has been achieved between the model and the experimental results.

A consistent behavior is observed for the thermal contribution to the static structure function, which dominates on short timescales. Its strongest signal emerges from the hot side of the cell and its weakest one from the cold side. The situation is more complicated for the solutal structure function. There, the generic behavior is a strong signal from the cold side and a weak signal from the hot side. This is consistent with the positive Soret coefficient of PS, which accumulates on the cold wall. For a dilute solution of a polymer with a high molar mass, the Soret coefficient is very large, resulting in a strong accumulation of the polymer on the cold side and a practically vanishing signal from the hot wall (see Fig. 4).

For a high concentration of a high polymer, however, the layer sequence no longer follows this generic ordering. The solutal signal can be inverted and assume its maximum value on the hot side, where the polymer concentration is reduced by the Soret effect. This is initially counter-intuitive and results from the combined effect of the concentration dependence of the concentration prefactor, the Soret coefficient, the diffusion coefficient, and the viscosity in these entangled polymer solutions.

In comparison with small molecules, the experiments presented in this work are characterized by typical polymer properties. Notably, these properties include entanglements in the semidilute regime, which result in a significant increase in viscosity, as well as large Soret coefficients in dilute solutions, which decrease markedly with increasing polymer concentration. Since the experiment only provides access to averaged structure functions with a broad underlying concentration distribution within the cell—which is closely related to a broad dispersion of the transport coefficients—it appears that an experimental determination of the Soret coefficient, for example, is not feasible with the strong temperature gradients employed here.

The PS/Tol system is also a binary glass former with significantly different glass transition temperatures for its two constituents. Approaching the glass transition along the concentration axis leads to an additional slowing down of all dynamics at high polymer concentrations above, say, $c = 0.5$. This concentration range has barely been reached in the present study and shall be addressed in future investigations. Pending microgravitational experiments within the framework of the GIANT FLUCTUATIONS project of the European Space Agency (ESA) will, e.g., allow to circumvent the complicated influence of the layer-dependent gravitational quench at small wavevectors

## Data Availability

Data are available upon reasonable request from the authors.
